# Liver Hydatid Cyst Rupture with Respiratory Distress During COVID-19 Pandemic

**DOI:** 10.5334/jbsr.2663

**Published:** 2022-05-06

**Authors:** Julián Moreno Rojas, Daniel Vas

**Affiliations:** 1 Hospital Clínic de Barcelona, ES

**Keywords:** Hydatid cyst, Hepatic hydatid disease, Rupture, Acute abdomen, Anaphylaxis

## Abstract

We present a case of a young male patient who was brought to the emergency room with abdominal pain, fever, dyspnea and hypoxemia, and was initially oriented as an acute COVID-19 pneumonia. A thoracoabdominal computed tomography (CT) was performed to rule out pneumonia and the abdominal extension of the study revealed a hepatic hydatid cyst with rupture into the abdominal cavity with respiratory distress secondary to an anaphylactic reaction that, if left untreated, may lead to a fatal outcome. An urgent laparotomy was performed with cyst resection. The follow-up and complications are briefly described.

## Summary

While the world is struggling with the pandemic of a beta-coronavirus known as severe acute respiratory syndrome coronavirus 2 (SARS-CoV-2), clinicians should have a low threshold for suspicion of COVID-19 (coronavirus disease-19) at the emergency room, thus it may present with a wide variety of symptoms and can lead to misdiagnosis during the outbreak.

We present a case of a young male patient who was brought to the emergency room with abdominal pain, fever, dyspnea, and hypoxemia, and was initially oriented as an acute COVID-19 pneumonia. A thoracoabdominal computed tomography (CT) was performed to rule out pneumonia and the abdominal extension of the study revealed a hepatic hydatid cyst with rupture into the abdominal cavity with respiratory distress secondary to an anaphylactic reaction that, if left untreated, may lead to a fatal outcome. An urgent laparotomy was performed with cyst resection. The follow-up and complications are briefly described.

## Background

Since December 2019, a novel strain of beta-coronavirus was documented to cause a cluster of cases of acute pneumonia with high transmissibility in Wuhan, China [1] and rapidly spread throughout the globe. By March 2020, the World Health Organization (WHO) announced that COVID-19 had reached pandemic status [2].

Among patients attending to emergency departments (EDs), prompt identification of those with suspected COVID-19 is important in order to avoid secondary transmissions. However, clinical diagnosis is challenging because the disease may present with wide variety of nonspecific symptoms such as myalgia, cough, or fever [3,4], and gastrointestinal symptoms have been also described in 23.6% of the cases [5].

As the prevalence of COVID-19 increases, clinicians should have a low threshold for suspicion of COVID-19 that, in some cases, may lead to misdiagnosis and delayed treatment of other medical conditions. Therefore, emergency physicians’ approach to atypical clinical presentations of COVID- 19 should facilitate early recognition and promptly trigger diagnostic procedures such as chest computed tomography (CT) in order to look for common imaging presentations of COVID-19 or possible differential diagnosis. We present a case of an adult male in his late twenties who was initially diagnosed with COVID-19 pneumonia instead of a systematic and thoracic manifestation of a potentially life-threatening abdominal disease, a direct rupture of a liver hydatid cyst.

## Case History

We present a case of a male patient of North African origin in his late twenties who was brought to the emergency department with four days of abdominal pain, fever, diarrhea, and dyspnea. There was no medical history of relevance. On arrival, the physical exam was significant for a temperature of 38.2ºC, heart rate of 128 beats/min, respiratory rate of 40 breaths/min, blood pressure of 90/60 mmHg, O2Sat of 87% at room air, and mild pain in the right upper quadrant without peritoneal irritation.

Lab work showed elevated D-dimer (3344 ng/dl), C-reactive protein (15 mg/L), and alkaline phosphatase (136.8 U/L). The chest X-ray depicted a right lower lobe opacity, accompanied by hypoxaemia (PaO2/FiO2 ratio 150); however, the COVID-19 RT-PCR test was negative.

**Figure 1:**
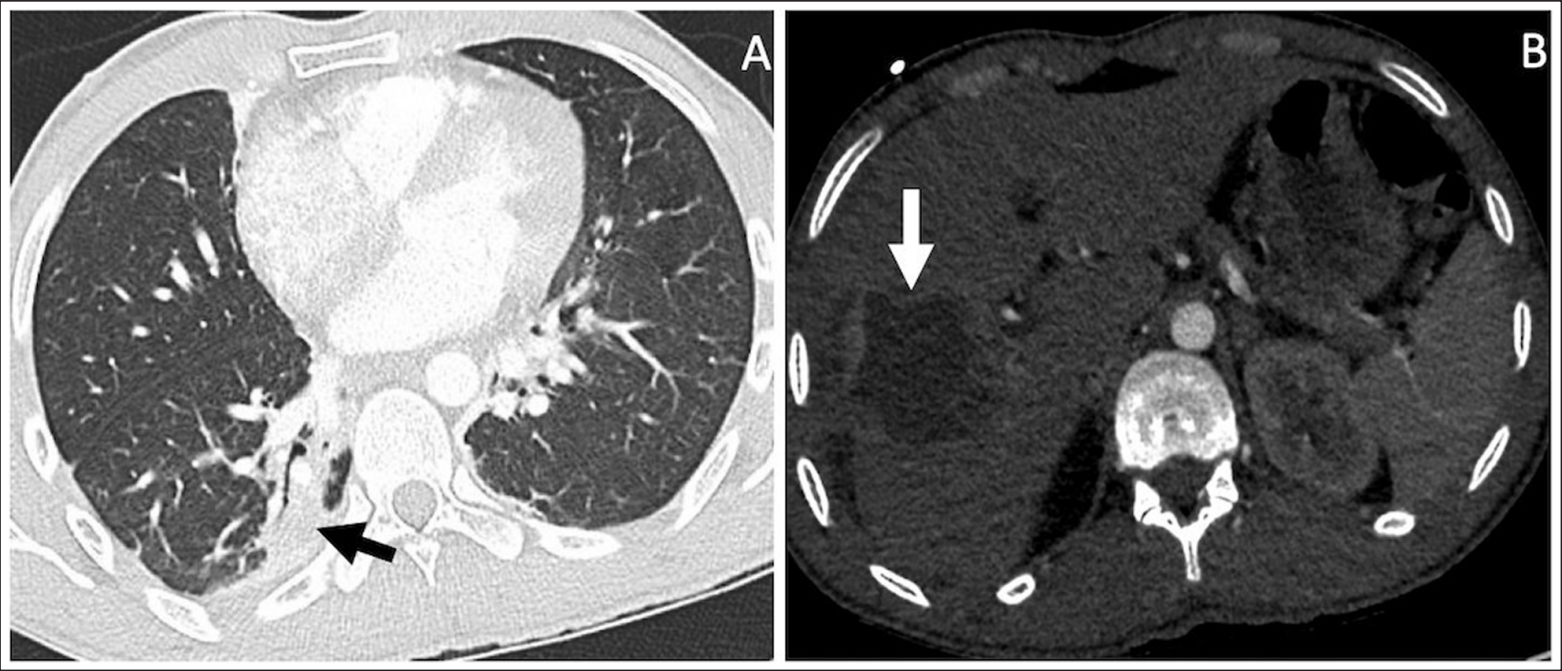
CT pulmonary angiogram (CTA) shows in **A** a right lower lobe atelectasis (black arrow), and in **B** a cystic liver lesion (white arrow).

Based on the initial workup, a severe pneumonia was suspected without ruling out COVID-19 infection, despite the negative RT-PCR test, thus the majority of ED visits at that moment of the pandemic was still due to this virus. A combination of antibiotic therapy was started, with ceftriaxone and azithromycin to cover multiple possible pathogens in the context of sepsis caused by the suspected community-acquired pneumonia (CAP). Hydroxychloroquine and lopinavir/ritonavir were added to the treatment to cover nonbacterial infections. Due to worsening of respiratory distress and hypotension, enoxaparin infusion was started, and a CT pulmonary angiogram was requested to rule out pulmonary embolism (PE). The chest imaging showed segmental atelectasis in the right lower lobe, without signs of pulmonary infection. Incidentally a cystic liver lesion was identified on the upper abdominal sections (***Figure 1***).

**Figure 2:**
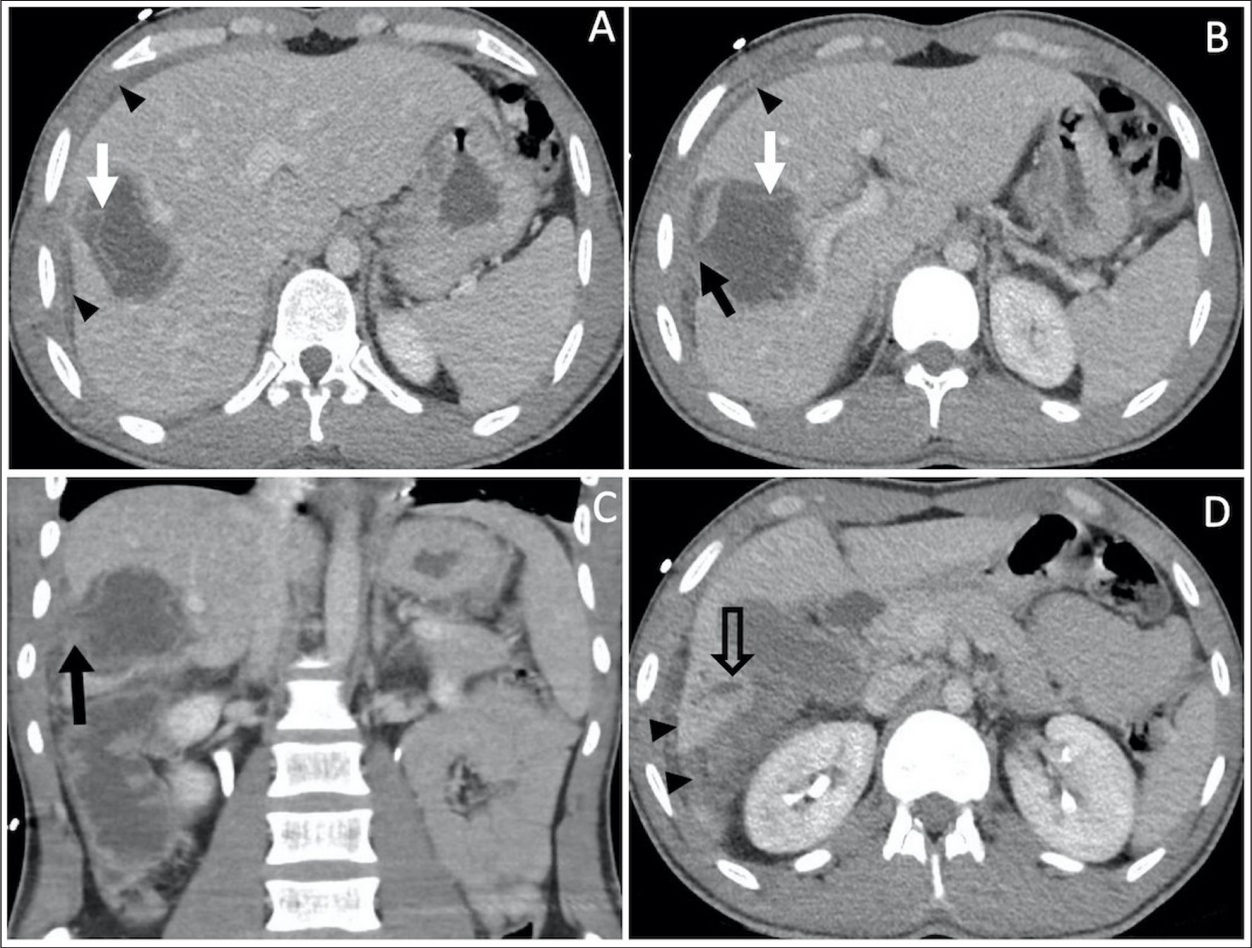
Contrast-enhanced abdominal CT shows a cystic liver lesion with a detached serpentine hyperdense membrane within the cyst, consistent with water lily sign (white arrow in **A** and **B**). There is a subcapsular wall defect (black arrow in **B** and **C**), fat stranding and free perihepatic fluid (arrowheads in **A**, **B** and **D**). Note the focal intrahepatic bile duct dilatation (hollow arrow in **D**).

Consequently, an abdomimal CT was performed, depicting a liver cyst in segment V/VI with a detached hyperdense membrane floating within the cyst. In a posterior subcapsular region a wall defect was observed, associated to a focal intrahepatic bile duct dilatation that reached the cyst. Fat stranding and a small amount of free perihepatic fluid were also present (***Figure 2***). These findings suggested a liver hydatid cyst with direct rupture into the abdominal cavity causing a possible anaphylactic reaction that may explain the respiratory and gastrointestinal symptoms. The focal bile duct dilatation was probably due to intrabiliary rupture of the cyst or as a result of direct compression of biliary branches.

## Differential Diagnosis

Numerous intra-abdominal and hepatic diseases may produce changes in the chest radiography. Over half of the patients presents elevated hemi-diaphragm, pleural effusion, atelectasis, or right lobar consolidation and may result in an inaccurate diagnosis of pneumonia [9].

The imaging findings of a hydatid cyst can have a variety of appearances depending on the stage of evolution, and maturity of the disease. In contained rupture, the inner layer (endocyst) detaches from the outer layer (pericyst), which results in a curvilinear structure or floating membranes within the pericyst, finding that is highly specific for hydatid disease [10]. On the other hand, a hypodense hepatic lesion on abdominal CT can be mistaken as a liver abscess. CT findings that may suggest the presence of a liver abscess usually include rim enhancement on arterial and portal venous phases, associated with perilesional hyperemia [11]. These characteristics were not present on admission CT.

## Treatment

The patient underwent exploratory laparotomy and the intraoperative findings confirmed the direct rupture of a liver hydatid cyst. Cystectomy with partial pericystectomy and cholecystectomy with intraoperative cholangiography were performed. A minor communication between the bile duct and the cyst cavity was sutured. As an extensive intra-abdominal infection was present, postoperative medical treatment included broad antibiotic coverage with meropenem, vancomycin with a subsequent de-escalation to cefotaxime and metronidazole, and an antihelmintic, albendazole was also added to cover the hydatid disease.

## Outcome and Follow-Up

The patient developed fever by the fifth postoperative day and an abdominal CT was performed to rule out postoperative complications, showing a perihepatic collection. A relaparotomy was performed and the surgical exploration revealed a fistulous communication between a bile duct and the cyst cavity, which was sealed with suture. An omental patch was used to close the hepatic defect. Subsequently, an endoscopic retrograde cholangiopancreatography (ERCP) was performed and due to bile leakage, a plastic biliary prosthesis was placed in the right hepatic duct. Thereafter, a fast postoperative recovery permitted the withdrawal of the prosthesis and the patient was discharged being asymptomatic at a five-month follow-up.

## Comment

The vast majority of patients attending an ED with fever, dyspnea, hypoxemia, and abdominal pain (as in our case) during the COVID-19 outbreak may indeed have COVID-19 pneumonia. As these are nonspecific symptoms, complementary imaging studies are required in order to confirm the suspicion or to detect findings that support an alternative diagnosis. In our case the initial chest radiography was abnormal, and the worsening respiratory symptoms pointed to a suspected PE, which was ruled out by CT pulmonary angiogram, allowing for the exclusion of a COVID-19 pneumonia diagnosis and revealing a cystic hepatic lesion.

More than 1/3 of patients with SARS-CoV-2 infection may present with dyspnea and concomitant gastrointestinal symptoms such as diarrhea, nausea, and anorexia [12]. Respiratory distress or respiratory symptoms are secondary to an abdominal disease and may be misdiagnosed as pulmonary origin. In the context of the COVID-19 pandemic, an unusual clinical presentation may require additional imaging studies to rule out an underlying abdominal disease.

Hydatid disease is a zoonotic parasitic infection caused by the Echinococcus tapeworm. Echinococcus granulosus is the most common cause of hydatid disease in humans, and it is a common infection worldwide. Humans are intermediate hosts, after accidentally ingesting parasitic eggs from infected animals [6,7]. The liver is the most commonly affected organ with cystic lesions that may have several complications such as intrabiliary rupture, cyst superinfection, direct rupture into the abdominal or thoracic cavities, or mass effect on adjacent structures [6,7]. Direct intra-abdominal rupture is more frequent in large, thin-walled hepatic hydatid cysts located near the liver surface. It can present clinically with acute abdomen and systemic anaphylactic reactions that may be life threatening [7,8].

The hydatic cyst is mainly found in the liver (75% of the cases), being asymptomatic in most cases [13]. The reported frequency of liver hydatid cyst rupture into the peritoneal cavity ranges from 1% to 16%. Rupture may result from trauma or may occur spontaneously from increased pressure of the cystic fluid [14].

There are three different types of hydatid cyst rupture: contained, communicating, and direct. In contained ruptures the pericyst remains intact and the endocyst is detached, which is seen in images as floating membranes within the cyst (water-lily sign), and it is usually due to degeneration, response to therapy or trauma [15]. A communicating rupture is characterized by passage of cyst contents to biliary radicals. Direct rupture occurs with both the endocyst and pericystic rupture, with the consequent passage of cyst content into a cavity [15]. The rupture into abdominal cavity can be a cause of anaphylactic shock and even death [7,16].

Errors in CT interpretation of hepatic cystic lesions could lead to misdiagnosis and erroneous management of a liver abscess, thus it consists in medical treatment and percutaneous drainage in certain circumstances [17,18], while the treatment of a liver hydatid cyst with direct rupture into the peritoneal cavity usually needs surgical intervention with complete resection and simultaneous treatment of fistulous tracts [13,19].

Our case underlines the importance of considering abdominal diseases in the differential diagnosis of COVID-19 pneumonia, especially when infrequent GI symptoms are present. It also highlights the key role of the radiologist in the characterization of a cystic hepatic lesion in order to differentiate correctly a complicated liver hydatid cyst from a liver abscess, as their therapeutic approach may be different. A complete medical history, an adequate clinical examination, and a correct interpretation of the imaging are fundamental for an accurate diagnosis.

To our knowledge this is the first published case of a complicated liver hydatid disease causing respiratory distress during the COVID-19 pandemic, leading to an initial orientation of a PE in the context of a suspected COVID-19 pneumonia. Due to these exceptional circumstances a complementary imaging study was able to depict correctly the underlying abdominal disease that required urgent surgical intervention.

## Teaching Points

More than 1/3 of patients with COVID-19 infection may present with dyspnea and gastrointestinal symptoms such as diarrhea, nausea, and anorexia. Liver hydatid cyst rupture into the peritoneal cavity can produce respiratory symptoms secondary to an anaphylactic reaction and need to be treated by surgical resection. There are three different types of liver hydatid cyst rupture; contained, communicating and direct.
